# Effect of Diluents on Mechanical Characteristics of Epoxy Compounds

**DOI:** 10.3390/polym14112277

**Published:** 2022-06-03

**Authors:** Anna Rudawska, Mariaenrica Frigione

**Affiliations:** 1 Faculty of Mechanical Engineering, Lublin University of Technology, Nadbystrzycka 36 St, 20-618 Lublin, Poland; a.rudawska@pollub.pl; 2 Department of Innovation Engineering, University of Salento, Via Arnesano, 73100 Lecce, Italy

**Keywords:** mechanical properties, epoxy resin, diluent

## Abstract

The aim of this work is to assess the influence of different commercial diluents on some mechanical properties of two bisphenolic epoxy compounds, cold-cured by a polyamide curing agent, to be employed as epoxy structural adhesives for building and industrial applications. The diluents under analysis were epoxy, bituminous, nitro, acrylic and extraction. The choice of these products was made on the basis of their wide commercial availability as diluents for epoxies used as adhesives and in different industrial and construction applications. The diluents were all added in small proportions, i.e., from 1 to 10 g per 100 g of epoxy resin. The cold-cured epoxy compounds were subjected to compressive (according to ISO 604) and static tensile (according to ISO 527-1) tests. The same mechanical tests were performed on both unmodified epoxy resins, for comparison purposes. On the basis of the obtained results, it was concluded that the influence of the presence of a diluent, and of its amount, on the mechanical properties of epoxy compounds depends on the type of resin and of diluent, as well as on the mechanical characteristics analyzed.

## 1. Introduction

Epoxy resins are currently widely employed in many different industrial sectors because of their ease of processing and curing, even at different temperatures, and extremely good mechanical properties [1,2,3,4]. These resins are polar by nature and, therefore, are able to develop good adhesion to various materials, such as plastics, concrete, glass, wood, most metals and ceramics [5,6]. During its hardening, a limited shrinkage can occur in the resin, which results in internal stresses. This inconvenience can be avoided with the addition of proper agents, such as additives or plasticizers [7,8,9,10,11,12,13]. After their complete curing, epoxy resins achieve considerable mechanical strength and resistance to chemicals and weathering [14,15,16,17,18,19,20,21]. Hardened epoxy resins are characterized by very good dielectric properties, high specific resistance and low dielectric loss factor [2].

Epoxy resins can be modified to obtain specific final characteristics: this can be done by adding proper modifiers depending on the property to be achieved [22,23]. In this regard, it is important to also select the appropriate resin and curing agent, as well as the adequate processing technology and curing parameters [10,24,25,26]. The common modifiers include fillers, diluents, plasticisers, antioxidants, dyes and pigments and stabilisers [27,28,29,30,31,32,33].

Highly viscous epoxy resins are difficult to process; this can represent an obstacle during their transformation to produce different components also containing fibers. To overcome this limitation, diluents can be introduced. The main characteristics of a diluent or a viscosity modifier for epoxy resins were presented by Monte [12] and Jagtap and More [34]. Lee et al. [28] underlined that epoxy resin diluents are able to improve some physical properties of the cured polymers (strength, elasticity and deformation), in addition to viscosity. Kregl et al. [7] demonstrated that the tensile strength and strain at break were improved in epoxy resins containing a diluent compared to the same epoxies containing a toughening agent. The properties that can be reached by resins, however, depend on the chemical nature and quantity of the diluent.

Non-reactive diluents are most commonly used since they do not affect the kinetics of the curing reaction of the resin [35]. Non-reactive diluents contain aromatic hydrocarbons, methacrylates and phthalates. Conversely, reactive diluents react with the epoxy mixture [8,36,37,38,39,40]. Reactive diluents can be divided into two groups, namely diluents with one or two epoxy groups, those composed of glycidyl ethers, liquid cycloaliphatic resins, olefin oxides and styrene oxide, and diluents that, while not containing any epoxide, are capable of reacting with other groups, such as unsaturated monomers, lactones and aliphatic nitriles. Filyanov et al. [41] investigated the influence of reactive diluents on the mechanical properties of a cured bisphenolic epoxy resin. They found that, with the addition of a highly reactive diluent (ETF-10), it was possible to obtain an epoxy with low viscosity in its uncured state without significantly reducing its mechanical properties. Montserrat et al. [42] confirmed that reactive diluents can be added to epoxy to reduce its viscosity, thus aiding its processability. Sinha et al. [33] investigated both non-reactive and reactive diluents on mechanical and thermal properties of bisphenol A epoxy resin. They reported that toluene (i.e., a non-reactive diluent) reduced the mechanical properties of the cured epoxy resin. In contrast, the addition of a reactive diluent (polyethylene glycol) significantly increased the tensile strength, hardness and fracture toughness of the cured epoxy compared to the reference resin.

Low-viscosity epoxy resins can be used to dilute diane-based epoxy resins, affecting their curing kinetics. Non-reactive diluents, on the other hand, do not generally reduce the reactivity of epoxies and can be, therefore, added in large amounts. Epoxy compounds containing diluents can be cold- or heat-cured; the properties of the obtained cured systems depend also on the type and amount of diluent added [36,38,43]. Ozgul and Ozkul [6] investigated the effect of epoxy resin, curing agent and diluent types on the properties of epoxy mortars. They noticed that the combination of various types of diluents and curing agents affected both the compressive and flexural strengths of the resulting epoxy mortars. Khalina et al. [29] investigated the effect of an aliphatic reactive diluent on the mechanical properties of different epoxy resins; the authors found that both the modulus and ultimate strength gradually declined as the diluent content was increased and, at the same time, the ductility steadily increased.

Even highly polar non-reactive diluents can affect the properties of cured epoxies; they are also known as anti-plasticizers. For example, upon the addition of 7–11 pph (parts per hundred) of a non-reactive diluent (e.g., tetrachlorethylene dibutyl phthalate) in a bisphenolic epoxy resin, an increase in its modulus of elasticity, hardness, tensile, flexural and compressive strength was recorded, but also reductions in elongation at break and impact strength.

Cured epoxy compounds containing different diluents were investigated in the present study. Two bisphenolic epoxy resins were selected, i.e., a standard epoxy and an epoxy modified with styrene. They were both cold-cured with a polyamide curing agent. Five different diluents (i.e., epoxy, bituminous, nitro, acrylic and extraction) were added to both resins in three amounts (1 g, 5 g and 10 g per 100 g of epoxy resin). The selected diluents are the most widely used to produce epoxy structural adhesives for building and industrial applications; they are also widely commercially available. The epoxy compounds, cured at ambient temperature, were subjected to compressive and static tensile tests to assess the effects of each diluent on the mechanical properties of both cured epoxies. The findings of the present study will be also of invaluable utility for the processing and industrial applications of such materials.

## 2. Materials and Methods

### 2.1. Epoxy Compounds

Thirty variants of cured systems, based on two epoxy resins modified with different diluents, were produced and analyzed. Two types of epoxy resins were employed: a standard bisphenolic epoxy resin, with the trade name Epidian 5, displaying an epoxy number of 0.48–0.51 mol/100 g, and a modified (with styrene) epoxy resin, with the trade name Epidian 53, with a minimum epoxy number of 0.41 mol/100 g. Both epoxy resins were supplied by Sarzyna Resins (Sarzyna Resins, Nowa Sarzyna, Poland).

Epidian 5 is an epoxy resin based on bisphenol A, which is the base resin widely used as an adhesive and matrix of composites for various applications. This epoxy displays a maximum viscosity of 3000 m·Pas (at 25 °C) and density at 20 °C of 1.17 g/cm^3^ (data reported on datasheet of Epidian 5).

Epidian 53 consists of a mixture of bisphenol A and epichlorohydrin. It is a styrene-modified epoxy resin (the amount of styrene ranging from 13 to 15 g per 100 g of resin). This resin has a lower viscosity (ranging from 900 to 1500 m·Pas at 25 °C) and a density of 1.11–1.15 g/cm^3^, measured at 20 °C. Epidian 53 resin has an average molecular weight of less than 700. The previous data was taken from the manufacturer’s resin data sheet.

Both resin systems were cold-cured upon the addition of a polyamide curing agent, indicated as PF (Sarzyna Resins, Nowa Sarzyna, Poland). This curing agent is characterized by an amine number of 350–400 mg KOH/g. Epoxy compounds were produced by mixing each epoxy resin with the PF curing agent in the ratio 100/50, as indicated by the supplier. The main properties and characteristics of the materials used in this study, i.e., the two epoxy resins and the polyamide curing agent, can be found in [18,44,45].

The epoxy compounds (i.e., the mixes composed by one of the two epoxy resins and the polyamide curing agent) are widely used in the production of adhesives to join different materials. The addition of a suitable diluent in the adhesive mix may bring some advantages, both technological (i.e., longer pot-life) and in terms of final performance.

In this work, the term “epoxy compound” indicates the mixture consisting of one of the selected epoxy resins and the hardener PF, with the possible addition of a diluent.

Five diluents were used in the investigation, namely epoxy, bituminous, nitro, acrylic and extraction. They were all provided by Dragon Poland Company (Dragon Poland Company, Cracow, Poland). All diluents are reported as low-viscosity substances, even though the exact values of their viscosity are not reported on their datasheet. Table 1 summarizes the data relative to their density, along with the label with which they were indicated in this study.

The diluents reported in Table 1 can be divided into two groups based on their chemical structures. In the hydrocarbon class, the following were chosen: epoxy diluent, bituminous diluent and extraction diluent. In the class of oxygen-containing diluents, on the other hand, there were nitro diluent and acrylic diluent.

The epoxy diluent can be effectively employed as diluent for different epoxy products because of a similar chemical nature. It has a low sulfur content and it does not contain benzene; in addition, it does not change the color of the diluted resin. The bituminous diluent is a mixture of hydrocarbons containing ethylbenzene, xylene, ethyl acetate and butyl acetate, while the nitro one is a mixture of ethanol and ethyl acetate. This latter has a low sulfur content and contains additives able to improve the flow (decreasing the viscosity) of epoxy paints and coatings; its addition does not lead to yellowing of the diluted resin. The acrylic diluent contains C8–C10 alkyl polyglucoside and ethyl acetate, with a low sulfur content. Finally, the extraction diluent contains low amounts of enzymes in hydrogen and hexane. Further information on the diluents used can be obtained from the technical data sheets of the materials, provided by the manufacturer of all products (Dragon Poland Compony, Skawina, Poland).

Each diluent was added in one of the two epoxy resin in three amounts, i.e., 1 g, 5 g and 10 g per 100 g of epoxy resin. Therefore, three variants for each couple of epoxy resin/diluent were produced, with a total number of 30 systems, whose compositions and designations are reported in Table 2 and Table 3 for the Epidian 5 and Epidian 53 epoxies, respectively. For comparison purposes, two reference epoxy compounds, based on the two epoxy resins not containing any diluent, were also produced and analyzed, namely EP5/PF 100/50 and EP58/PF 100/50. 

### 2.2. Preparation and Curing of the Epoxy Samples

Samples of the epoxy compounds suitable for compression and tensile tests according to ISO 604 and ISO 527-1 standards, respectively, were produced.

The resin, curing agent and diluent of each epoxy compound, in the amounts reported in Table 2 and Table 3, were mixed and mechanically stirred with the aid of an anchor mixer, operating at 460 rpm for 2 min. The gas bubbles formed in each mixture during this process were then removed with a vacuum pump for additional 2 min at laboratory temperature. Before the pouring of each epoxy compound, the surfaces of the molds were sprayed by Polisiform coating (Polish Silicones, Nowa Sarzyna, Poland) to facilitate the removal of the cured products from the molds. In the case of cylindrical samples, during the curing process an additional degassing step was performed: the gas bubbles were, in fact, removed using a suitably modified mold equipped with a degassing hole.

**Figure 1:**
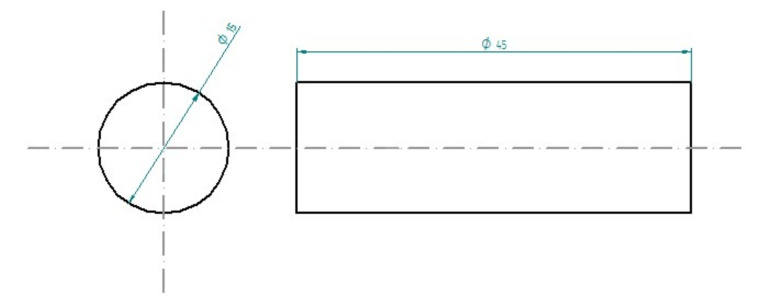
Dimensions of the cylindrical samples.

**Figure 2:**
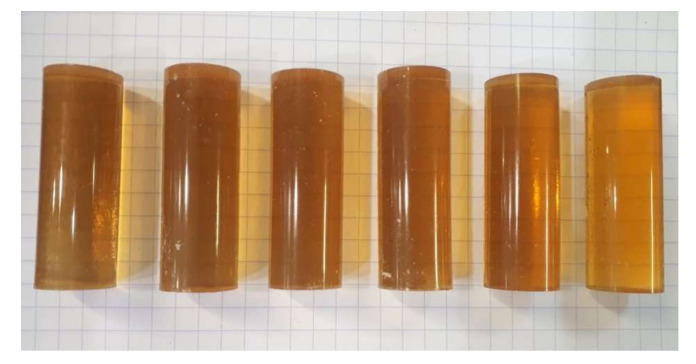
Cylindrical samples of epoxy.

The specimens for compressive tests were fabricated in cylindrical shape, as illustrated in Figure 1 and Figure 2. Their dimensions were: diameter 15 mm and height 45 mm. For each composition, six specimens were produced.

**Figure 3:**
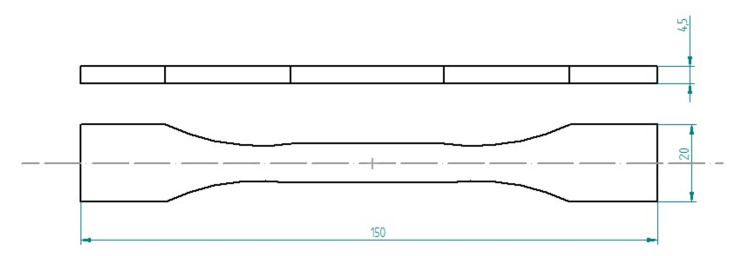
Dimensions of a dog-bone sample.

**Figure 4:**
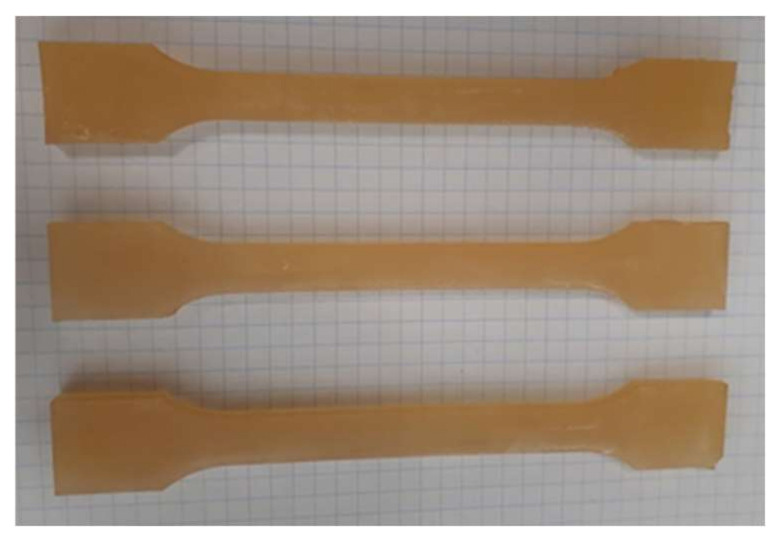
Dog-bone specimens of epoxy.

For the tensile tests, six dog-bone samples for each compound, according to the compositions reported in Table 2 and Table 3, were produced. These samples are illustrated in Figure 3 and Figure 4.

All the specimens were produced by casting the liquid mixtures in molds of proper shape and dimensions: the cylindrical samples were fabricated employing polyethylene molds of 10 mL capacity while the dog-bone specimens were prepared in silicone molds. All the specimens were cured at 22 °C and 22 (±1)% R.H. for 14 days. 

After the curing process, the specimens were milled (spindle rotation 850 rpm, feed rate 74 m/min) to obtain flat and perpendicular surfaces to assure a correct clamping of the specimens in the grip on the testing machine. The milling was performed with a FNB26-type milling machine (AVIA, Warsaw, Poland). 

### 2.3. Mechanical Tests

Mechanical tests were performed on the produced specimens with the aid of a Zwick/Roell Z150 testing machine (ZwickRoell GmbH&Co. KG, Ulm, Germany). From the results of such experiments, it was possible to compare the mechanical behavior of the epoxy compounds, assessing the effects of the different solvents added in diverse amounts.

Cylindrical samples (Figure 2) were subjected to compressive tests according to the ISO 604 standard. The tests were performed at 10 mm/min, employing a preload force of 100 N, with a speed of the test module equal to 1 mm/min. From compression tests, it was possible to measure the compressive modulus, Ec (MPa), and the compressive strength, σ_M_ (MPa).

The mechanical tests in tensile mode were performed according to ISO 527-1 standard on dog-bone specimens, shown in Figure 4. The tensile tests were performed with a preload of 0.1 MPa and using a rate of 20 mm/min. Tensile modulus, Et (MPa) and strength σ_t_ (MPa) were recorded during the tests.

## 3. Results

### 3.1. Mechanical Tests in Compression Mode

#### 3.1.1. Influence of Diluents on the Compressive Modulus, Ec, of Epoxy Compounds

**Figure 5:**
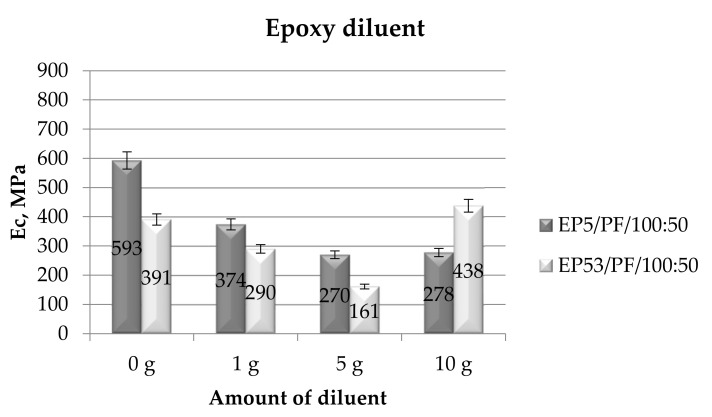
Compressive modulus calculated for Epidian 5 and Epidian 53 epoxy compounds as a function of the amount of epoxy diluent.

The effect of the selected diluents, added in different amounts, on the mechanical characteristics on the two epoxy resins under investigation, i.e., Epidian 5 and Epidian 53, was first assessed by performing the tests in compression mode. The compressive modulus values measured on epoxy compounds upon the addition of epoxy diluent are illustrated in Figure 5. For comparison purposes, the compressive modulus values recorded on each of the pristine resins are also presented.

The highest value of compressive modulus was observed for the reference Epidian 5 bisphenolic epoxy resin, i.e., the EP5/PF system. The epoxy resin modified with styrene, i.e., Epidian 53, displayed an appreciably lower compressive modulus, probably because of the lower epoxy number of this latter resin, and mainly to its modification with styrene. When the epoxy diluent was added in Epidian 5 resin, an appreciable decrease (more than halved) in compressive modulus was recorded, particularly at greater (5 g and 10 g) amounts. The data obtained for Epidian 53 resin with the epoxy diluent are generally lower than the values measured on formulations prepared with Epidian 5 epoxy, with the exception of EP53/PF system containing the largest amount of this diluent. Excluding this latter, the effect of the epoxy diluent is again revealed in a steady reduction in the compressive modulus by increasing the amount of the diluent. An unexpected behavior was observed for EP53/PF/REP 100:50:10 system, with a value of compressive modulus even greater than that measured for the unmodified resin. The standard deviation calculated for the results of these tests was in all cases not greater than 10–11%, testifying to a good reproducibility of results.

**Figure 6:**
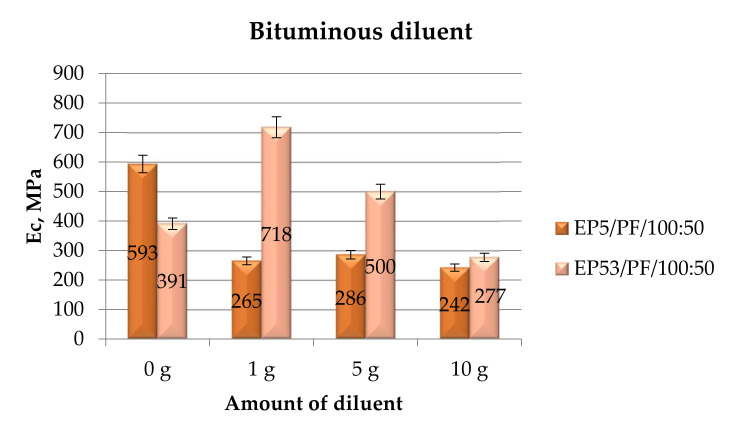
Compressive modulus calculated for Epidian 5 and Epidian 53 epoxy compounds as a function of the amount of bituminous diluent.

Figure 6 illustrates the data relative to the compressive modulus recorded on the epoxy compounds based on Epidian 5 and Epidian 53 resins unmodified and modified with bituminous diluent.

The addition of this diluent affects in different ways the compressive modulus of the two investigated resins. It produces an appreciable decrease in this property in the case of Epidian 5 epoxy resin, even at very low amounts (1 g and 5 g) of diluent. The influence of the diluent content, in fact, is almost negligible for this resin. On the other hand, the higher compressive moduli were obtained for EP53/PF/RB epoxy compounds at the lower contents of bituminous diluent, i.e., 1 g and 5 g. These values are even greater than that measured on unmodified Epidian 53 epoxy. When adding a greater amount of bituminous diluent to this resin, on the other hand, a sharp drop in compressive modulus is recorded, which falls to a value similar to that recorded for the other resin containing the same amount of the diluent. In addition, in these tests, a standard deviation never exceeding 10% was calculated. 

**Figure 7:**
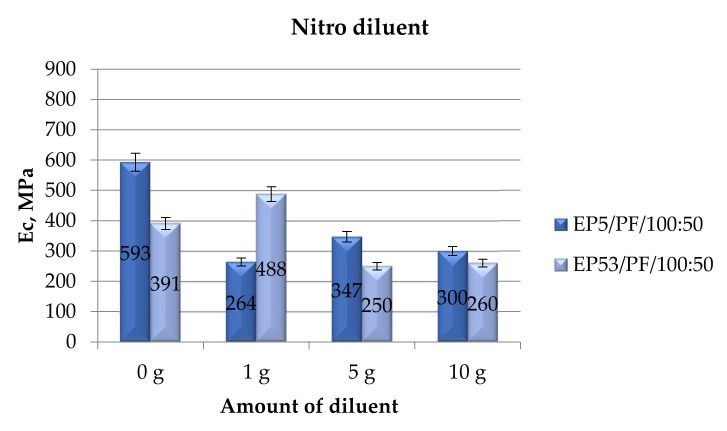
Compressive modulus calculated for Epidian 5 and Epidian 53 epoxy compounds as a function of the amount of nitro diluent.

When nitro diluent was added in both epoxy resins, the compressive modulus values reported in Figure 7 were recorded.

A different trend was observed for the compressive modulus measured on Epidian 5 and Epidian 53 resins, respectively, containing nitro diluent in increased amounts. A significant decrease in this property (more than halved with respect to the value calculated on the unmodified resin) was recorded for Epidian 5 epoxy at the lowest content of nitro diluent. At greater diluent contents, a slightly greater value of compressive modulus was recorded that, however, remains well below that measured on Epidian 5 epoxy. When the same diluent in the lowest amount (1 g) was added to Epidian 53 epoxy resin, a compressive modulus 25% greater than that measured on the pristine resin was measured. Conversely, by increasing the content of nitro diluent, an appreciable drop in this property was recorded, being the differences between 5 g and 10 g contents almost negligible, taking into account the standard deviation of results.

**Figure 8:**
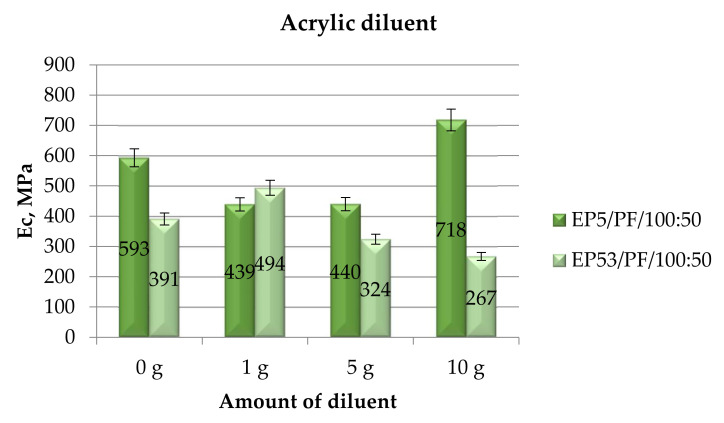
Compressive modulus calculated for Epidian 5 and Epidian 53 epoxy compounds as a function of the amount of acrylic diluent.

The moduli calculated in compressive mechanical tests performed on epoxy compounds, unmodified and modified with acrylic diluent, are presented in Figure 8. The negative influence of the addition of a diluent on the compressive modulus of epoxy systems based on Epidian 5 resin is again confirmed in the case of acrylic diluent, although at the lowest amounts (1 g and 5 g). On the contrary, when this diluent was added to Epidian 5 epoxy in the greatest amount (10 g), an appreciable increase (above 20%) in the compressive modulus was recorded. The results reported in Figure 8 proved a different effect of the same diluent on the compressive modulus of Epidian 53 epoxy, the higher the diluent content, the lower the modulus. Furthermore, for this latter epoxy resin, upon addition of the smallest amount of acrylic diluent, a compressive modulus greater (+26%) than that measured on unmodified Epidian 53 resin was measured. 

**Figure 9:**
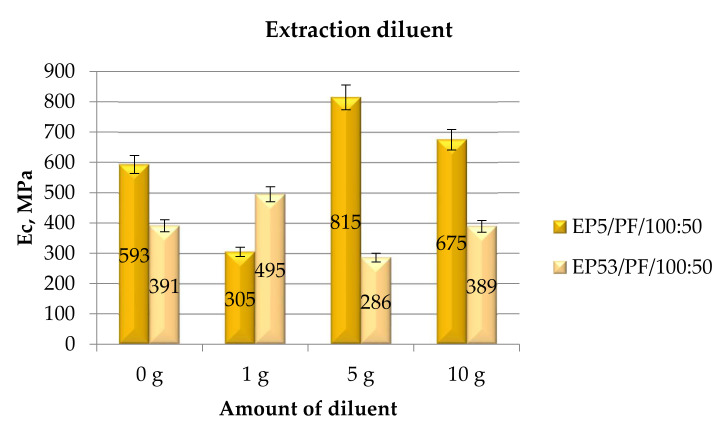
Compressive modulus calculated for Epidian 5 and Epidian 53 epoxy compounds as a function of the amount of extraction diluent.

Finally, in Figure 9 the compressive modulus measured on epoxy compounds containing different amounts of extraction diluent are reported. The compressive modulus of compounds based on Epidian 5 resin experienced a sharp decrease at the lowest diluent content with respect to unmodified epoxy. On the other hand, by increasing extraction diluent content, this property grew again, achieving much greater (+37% and +14% at 5 g and 10 g of extraction diluent, respectively) values than that of the pristine resin. The same diluent had an opposite influence on the compressive modulus of Epidian 53 resin: an increase in this property (+27%) when 1 g of extraction diluent was added; at greater diluent contents, the modulus experienced a limited decrease, until reaching the same value calculated for the unmodified resin at 10 g of diluent. Once again, the standard deviation of data was generally never greater than 10–15%.

From this attentive analysis, it was concluded that the effect of the addition of the different diluents on compressive modulus of the epoxy compounds, based on two epoxy resins, depended on the kind of resin and on the type of diluent added to each epoxy. Generally speaking, the diluents had a detrimental effect on this property when analyzing Epidian 5 resin, i.e., a standard bisphenol A epoxy resin, characterized by a higher value of compressive modulus in its unmodified formulation. The only exceptions were represented by the acrylic and extraction diluents, whose addition in moderate amounts led to enhanced compressive modulus. Referring to the styrene-modified epoxy, i.e., Epidian 53, the diverse diluents differently affected its compressive modulus without a clear trend. It seemed, moreover, that the diluents were able to increase to a certain extent (depending on the kind of diluent) the compressive modulus of this resin but only at the lowest diluent content, with the exception of epoxy diluent.

#### 3.1.2. Influence of Diluents on the Compressive Strength, σ_M_, of Epoxy Compounds

**Figure 10:**
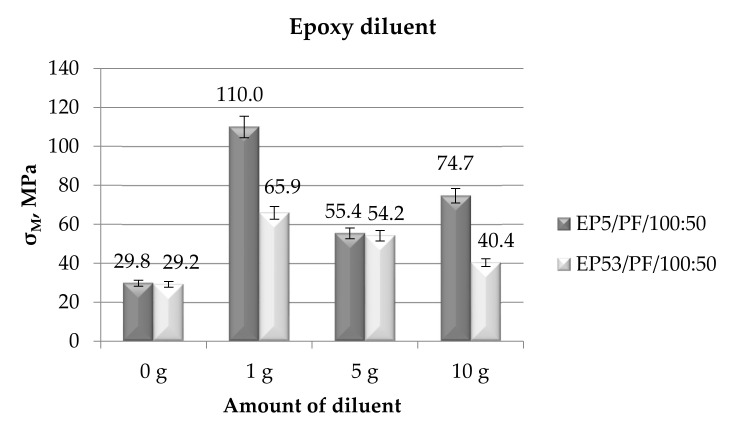
Compressive strength calculated for Epidian 5 and Epidian 53 epoxy compounds as a function of the amount of epoxy diluent.

The mechanical tests, carried out in compression mode on Epidian 5 and Epidian 53 epoxies modified by the different diluents, allowed us to assess the influence of these modifiers on the compressive strength of both resins. Figure 10 presents the compressive strength values measured on the epoxy compounds upon the addition of the first selected product, i.e., the epoxy diluent.

Similar values of the compressive strength were measured for both epoxies, i.e., the Epidian 5 and Epidian 53 resins. The effect of the epoxy diluent was more evident for the Epidian 53 epoxy, leading to generally improved values of its strength. No clear trend can be attributed to the content of the diluent in this resin: the strength is almost four times greater than that found on the unmodified resin at the lowest (1 g) diluent content; it is nearly doubled at 5 g of diluent and it rises again (+150%) at the greatest selected content. More limited was the growth in compressive strength measured on Epidian 53 resin upon the addition of the epoxy diluent—the greater the amount of solvent, the lower the compressive strength. In addition, in the case of the compressive strength data, the standard deviation of results never exceeded 10%. 

**Figure 11:**
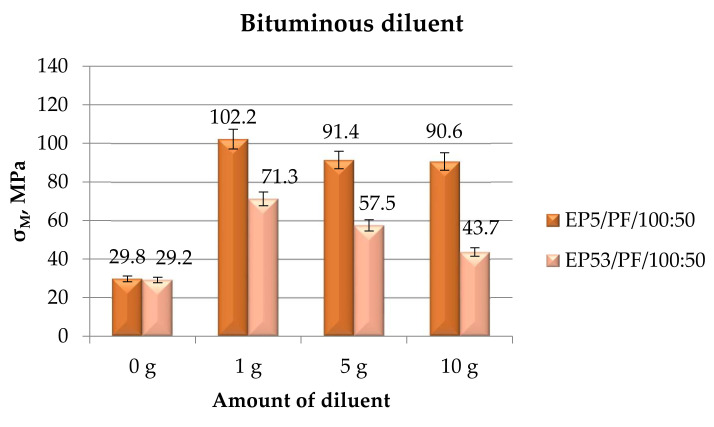
Compressive strength calculated for Epidian 5 and Epidian 53 epoxy compounds as a function of the amount of bituminous diluent.

Figure 11 shows the compressive strength calculated for EP5/PF and EP53/PF epoxy compounds, unmodified and modified with bituminous diluent.

In addition, in the case of bituminous diluent, its addition leads to an increase in compressive strength compared to the respective unmodified resins, i.e., Epidian 5 and Epidian 53. Again, the growth in strength was more marked for Epidian 5 resin, σ_M_ achieving the greatest value at the lowest modifier content. A general decrease in compressive strength was observed as the amount of bituminous diluent increased, irrespective of the resin type.

**Figure 12:**
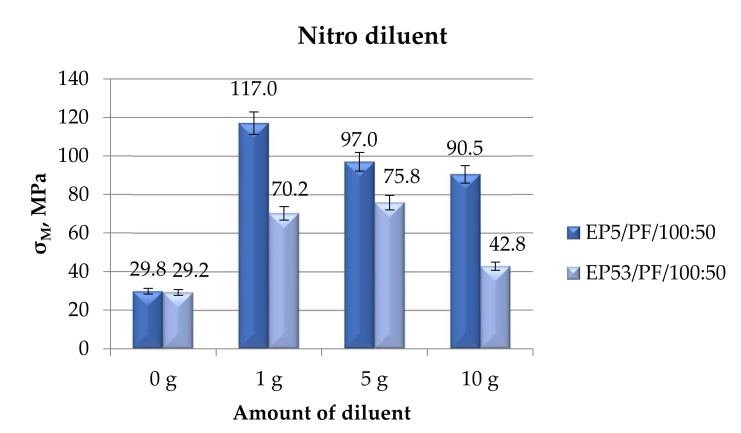
Compressive strength calculated for Epidian 5 and Epidian 53 epoxy compounds as a function of the amount of nitro diluent.

The comparison of the strength data obtained from mechanical compressive tests performed on epoxy compounds, with or without nitro diluent, is shown in Figure 12.

The compressive strengths recorded on compounds based on both epoxy resins, i.e., Epidian 5 and Epidian 53, were in line with what was previously observed: a substantial increase in compressive strength upon the addition of the diluent, more significant for Epidian 5 epoxy. This increase was generally reduced by raising the diluent content, with the exception of systems based on Epidian 53 epoxy. In this epoxy, in fact, the highest value of compressive strength was calculated when 5 g of nitro diluent were added.

**Figure 13:**
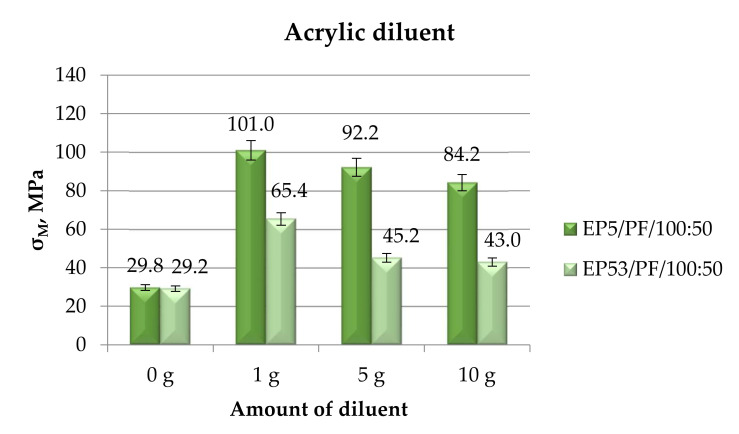
Compressive strength calculated for Epidian 5 and Epidian 53 epoxy compounds as a function of the amount of acrylic diluent.

Figure 13 presents the values of compressive strength measured on epoxy compounds, based on Epidian 5 and Epidian 53 epoxy resins, modified with different amounts of acrylic diluent.

The trend observed for the compression strength values that were due to the addition of acrylic diluent in different amounts was substantially similar to what already observed for the other modifiers. It is confirmed, therefore, that the addition of a diluent modifier brings about an increase in compressive strength to both epoxies, and in particular to Epidian 5 epoxy resin, this growth being reduced at increased diluent contents.

**Figure 14:**
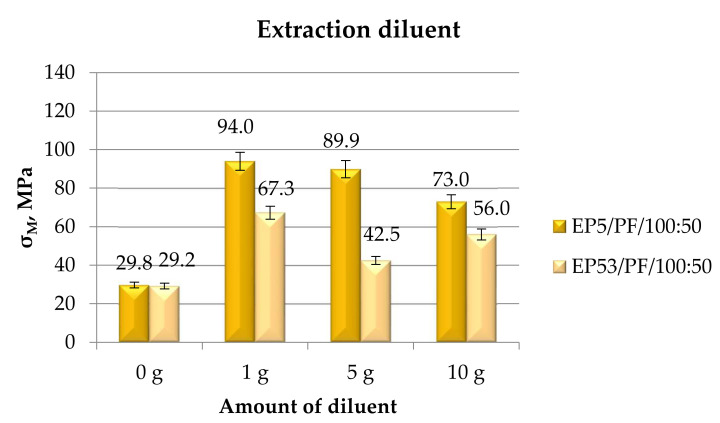
Compressive strength calculated for Epidian 5 and Epidian 53 epoxy compounds as a function of the amount of extraction diluent.

Similar conclusions can be drawn by the observation of the results, in terms of strength, of the compressive tests performed on the epoxy compounds unmodified and modified with extraction diluent, as shown in Figure 14.

Accordingly, the extraction diluent produced a noticeable increase in the compressive strength of Epidian 5 resin; by increasing the amount of this diluent, the strength value declined, although still remaining well above the value measured for pristine Epidian 5. Similarly, the compounds based on Epidian 53 epoxy experienced a growth in the compressive strength upon the addition of the extraction diluent. The lowest increase (around 45%) was in this case observed when 5 g of diluent were added.

In conclusion, it is possible to attribute a positive role to all diluents analyzed, all being able to improve, even if to different extents, the compressive strength of both epoxy resins under investigation. Generally speaking, the best enhancements can be achieved for the bisphenolic Epidian 5 resin at the lowest (1 g) diluent content, this last occurrence representing an advantage from an operative point of view. At this content, Epidian 5 epoxy resin achieved compressive strengths four times greater than the strength measured on the unmodified resin, i.e., about 117 MPa. Epidian 53 epoxy, on the other hand, displayed the highest compressive strength value of about 76 MPa upon addition of 5 g of nitro diluent.

### 3.2. Mechanical Tests in Tensile Mode

#### 3.2.1. Influence of Diluents on the Tensile Modulus, Et, of Epoxy Compounds

**Figure 15:**
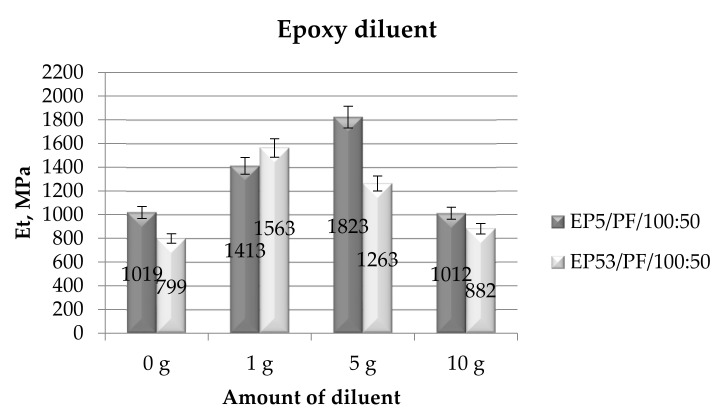
Tensile modulus calculated for Epidian 5 and Epidian 53 epoxy compounds as a function of the amount of epoxy diluent.

The investigation continued, then, to analyze the effects of the chosen diluents on the tensile mechanical properties of Epidian 5 and Epidian 53 epoxy resins. The results of the performed tests in terms of tensile modulus are first presented. In Figure 15, the data relative to the epoxy compounds, based on Epidian 5 and Epidian 53 epoxies, unmodified and modified with the epoxy diluent, are compared.

Comparing first the tensile modulus of the unmodified resins, it can be noticed that Epidian 5 epoxy resin offers a greater modulus with respect to Epidian 53, even testing both materials in tensile mode. This result can be again justified by the lower epoxy number of Epidian 53 resin, leading to a lower cross-linking density with respect to Epidian 5 epoxy and, in turn, to a lower stiffness. The modification of a bisphenolic epoxy resin with styrene, thus producing Epidian 53 resin, may also have contributed to the lower tensile modulus measured for this resin. Nevertheless, a very high stiffness value results in an excessive fragility of the epoxy, which is unsuitable for some applications. The data shown in Figure 15 allowed us to conclude that the addition of the epoxy diluent led to a general increase in the tensile modulus in both resins, especially at lower diluent contents, i.e., 1 g for Epidian 53 resin and 5 g for Epidian 5 epoxy. At the greatest amount of epoxy diluent (10 g), the effect of this diluent was rather limited. A good reproducibility of results can be deduced from the standard deviation values, always lower than 10%.

**Figure 16:**
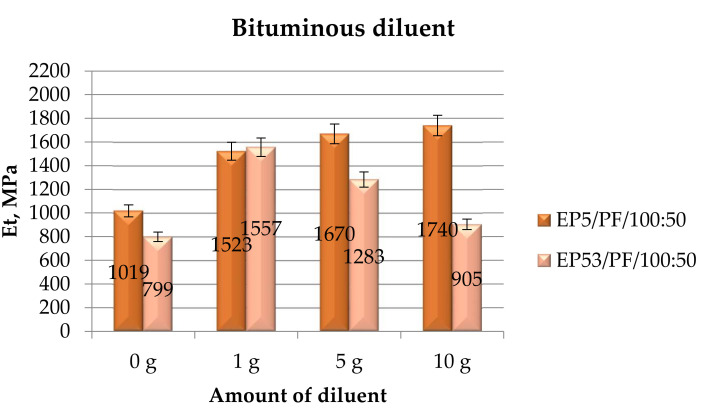
Tensile modulus calculated for Epidian 5 and Epidian 53 epoxy compounds as a function of the amount of bituminous diluent.

The tensile moduli calculated on epoxy compounds based on Epidian 5 and Epidian 53 resins, unmodified and modified with bituminous diluent, are presented in Figure 16.

In addition, the bituminous diluent was able to improve the tensile modulus of both resins, irrespective of its content in the mixtures. In the case of Epidian 5 epoxy compounds, the greater the content of the bituminous diluent, the higher the tensile modulus value, at least in the range of compositions analyzed. Conversely, the formulations based on Epidian 53 resin displayed values of tensile modulus that decreased by increasing the amount of diluent, still remaining greater than the same characteristic measured on the pristine resin. 

**Figure 17:**
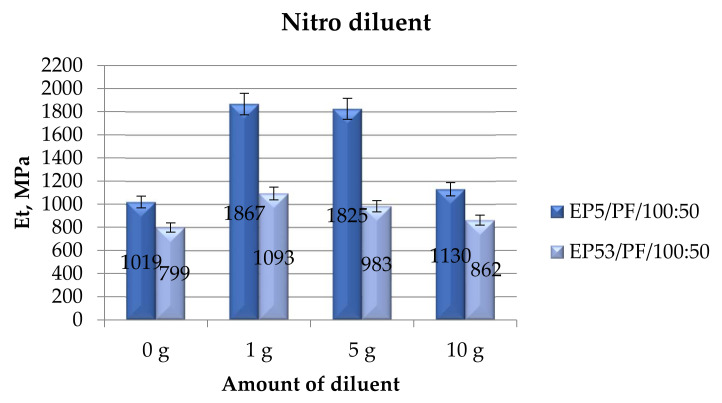
Tensile modulus calculated for Epidian 5 and Epidian 53 epoxy compounds as a function of the amount of nitro diluent.

The tensile modulus values recorded on Epidian 5 and Epidian 53 epoxy compounds, unmodified and modified with nitro diluent, are shown in Figure 17. 

As for the previously analyzed diluents, a general increase in the tensile modulus was recorded upon the addition of nitro diluent to Epidian 5 and Epidian 53 resins. The addition of greater amounts of nitro diluent caused a gradual decrease in the tensile modulus measured on compounds based on Epidian 53 resin. The higher values of this characteristic were obtained in compounds based on Epidian 5 epoxy containing 1 g and 5 g of nitro solvent. Beyond this composition, the tensile modulus of such systems experienced a strong reduction, nonetheless remaining greater (+11%) than that of the unmodified Epidian 5 resin.

**Figure 18:**
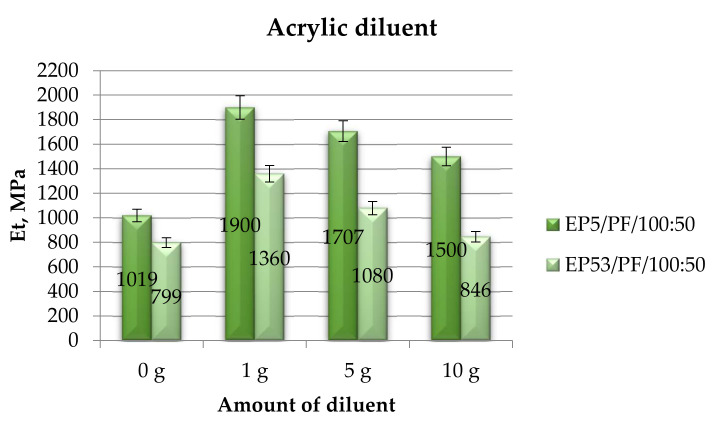
Tensile modulus calculated for Epidian 5 and Epidian 53 epoxy compounds as a function of the amount of acrylic diluent.

Figure 18 compares the tensile moduli obtained from the investigations performed on the Epidian 5 and Epidian 53 epoxy compounds, unmodified and modified with acrylic diluent. 

The addition of acrylic diluent produced an increase in tensile modulus, irrespective of the kind of epoxy resin. A basically constant decline in tensile modulus was recorded by increasing the amount of this diluent, from 1 g to 10 g, in both resins. The epoxy compounds based on Epidian 5 resin always displayed much greater values of tensile modulus with respect to those calculated on systems based on Epidian 53 epoxy. Standard deviation values were, also in this case, smaller than 10%. 

**Figure 19:**
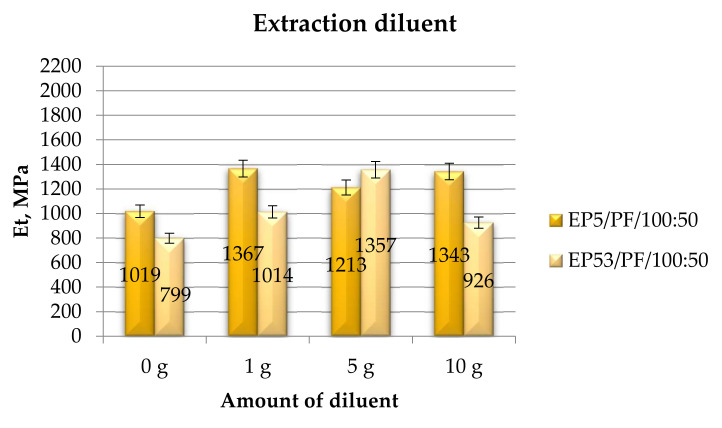
Tensile modulus calculated for Epidian 5 and Epidian 53 epoxy compounds as a function of the amount of extraction diluent.

Finally, in Figure 19, the tensile modulus measured on epoxy compounds, based on Epidian 5 and Epidian 53 resins, unmodified and modified with extraction solvent, are presented. The positive effect of diluents in increasing the tensile modulus was here confirmed for the extraction diluent. The influence of its amount, on the other hand, appeared different from what was observed on the other diluents. The compounds based on Epidian 5 epoxy resin displayed similarly high values of tensile modulus upon addition of 1 g and 10 g of extraction diluent. Conversely, the highest modulus measured on the compounds based on Epidian 53 epoxy resin was obtained upon the addition of 5 g of diluent. Adequate values of standard deviation of results assured that the data were reliable and reproducible.

From the analysis and comparison of the modulus data calculated in the tensile tests performed on the different systems, the following conclusions can be drawn: (i) the addition of diluents in compounds based on both Epidian 5 and Epidian 53 epoxies brought about general improvements in their tensile modulus, irrespective of the epoxy resin and the kind of diluent; (ii) albeit with some exceptions, the greatest modulus values were obtained at low quantities (1–5 g) of each diluent; and (iii) taking into account a few exceptions, the compounds based on the stiffer resin, i.e., Epidian 5 epoxy, displayed generally higher values of tensile modulus than those based on the less stiff Epidian 53 epoxy, irrespective of the type and content of the diluent.

#### 3.2.2. Influence of Diluents on the Tensile Strength, σ_t_, of Epoxy Compounds

**Figure 20:**
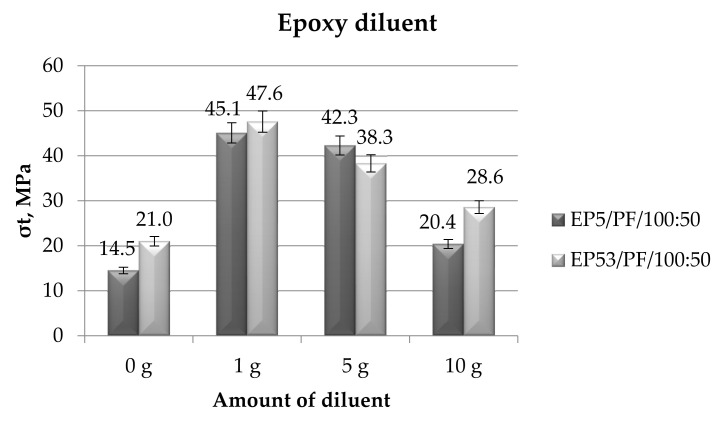
Tensile strength calculated for Epidian 5 and Epidian 53 epoxy compounds as a function of the amount of epoxy diluent.

The mechanical tests performed in tensile mode allowed us to also measure the tensile strength values as a function of the kind and the amount of diluents added to the Epidian 5 and Epidian 53 epoxies. In Figure 20, the data relative to the epoxy compounds based on Epidian 5 and Epidian 53 resins, unmodified and modified with the epoxy diluent, are first presented.

The examination of the results of tensile tests revealed that Epidian 53 epoxy resin offers a greater tensile strength (+45%) with respect to Epidian 5 epoxy resin. The value of the tensile strength of this latter, i.e., a bisphenolic epoxy resin cured at 22 °C, is in line with the tensile strength reported for a two-part system composed by a bisphenolic epoxy resin cold cured by a polyamide, i.e., 12 MPa [46].

While continuing to analyze the effects of the epoxy diluent on the tensile strength of the compounds based on the two epoxy resins, it was noticed that this diluent produced an increase in this characteristic, irrespective of the kind of epoxy. The rise in strength was greater at lower diluent contents (i.e., 1 g and 5 g) for both resins; this characteristic was reduced by increasing the amount of diluent in both epoxy compounds. At a content of 5 g of diluent, the strength measured on Epidian 5 epoxy resin exceeds that calculated on Epidian 53. A standard deviation not greater than 15% testified a good reproducibility of results obtained from these experiments.

**Figure 21:**
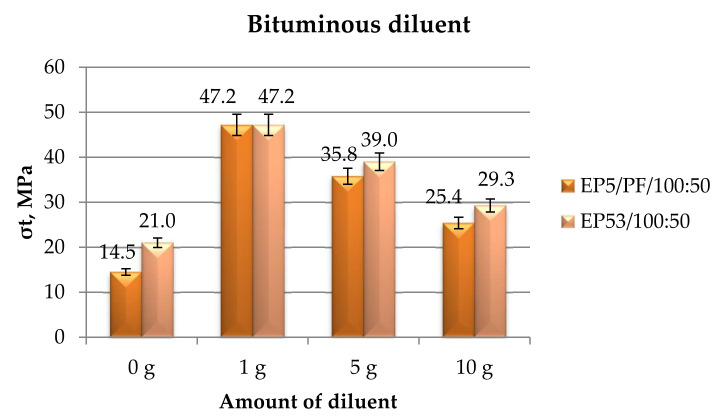
Tensile strength calculated for Epidian 5 and Epidian 53 epoxy compounds as a function of the amount of bituminous diluent.

The diagram reported in Figure 21 shows the average tensile strength values measured on epoxy compounds based on Epidian 5 and Epidian 53 epoxies, unmodified and modified with bituminous diluent in different percentages.

A clear trend was observed in this case, i.e., a growth in tensile strength compared to unmodified epoxy resins, which was reduced by increasing the diluent content. At the lowest amount of bituminous diluent, i.e., 1 g, very similar tensile strength values were measured on both epoxy resins. At greater diluent contents, the compounds based on the more resistant resin, i.e., Epidian 53, displayed higher tensile strength with respect to the epoxy systems based on Epidian 5 resin.

**Figure 22:**
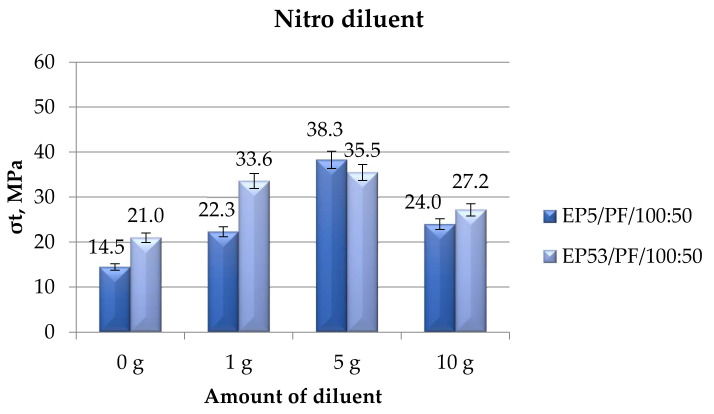
Tensile strength calculated for Epidian 5 and Epidian 53 epoxy compounds as a function of the amount of nitro diluent.

The comparison between tensile strength values calculated on epoxy compounds based on Epidian 5 and Epidian 53 epoxies, unmodified and modified with nitro diluent, is shown in Figure 22.

The highest tensile strength values were found when 5 g nitro diluent were added to both resins; at this concentration, the compound based on Epidian 5 epoxy resin was able to display a greater strength over that based on the more resistant Epidian 53. The tensile strength declined at the lowest (1 g) and greatest (10 g) amounts of nitro diluent, remaining for both epoxies above the values measured on the pristine resins.

**Figure 23:**
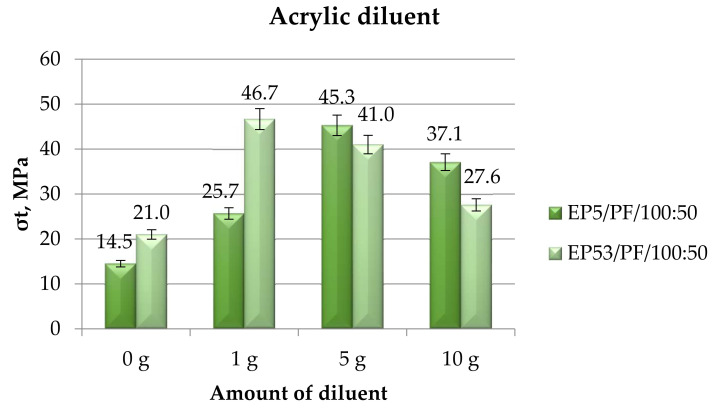
Tensile strength calculated for Epidian 5 and Epidian 53 epoxy compounds as a function of the amount of acrylic diluent.

The results, in terms of strength, of the tensile mechanical tests performed on Epidian 5 and Epidian 53 epoxy compounds, unmodified and modified with acrylic diluent, are presented in Figure 23.

In line with previous results, the addition of acrylic diluent produced a general increase in tensile strength with respect to both unmodified resins. Referring first to the compounds based on Epidian 53 epoxy resin, the beneficial effect in terms of strength growth was steadily reduced by increasing the diluent content. What is different is the influence of the acrylic diluent content on Epidian 5 epoxy resin: the greatest value in strength was achieved at a 5 g content of this diluent. This value was even greater than that measured on the compound based on the more resistant Epidian 53 epoxy at the same diluent content. At the greatest (10 g) and especially at the lowest (1 g) amounts of acrylic diluent, compounds based on Epidian 5 epoxy displayed lower tensile strength values.

**Figure 24:**
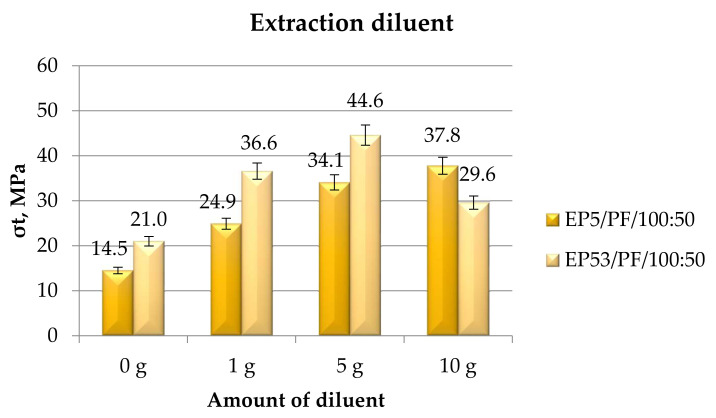
Tensile strength calculated for Epidian 5 and Epidian 53 epoxy compounds as a function of the amount of extraction diluent.

In Figure 24, the tensile strength measured on Epidian 5 and Epidian 53 epoxy compounds are presented as a function of the content of extraction diluent.

The positive effect on tensile strength that was due to the addition of a diluent in both epoxies was confirmed also in the case of the extraction diluent. The increase in tensile strength was simultaneous with the increase in the amount of this diluent in the case of the systems based on Epidian 5 epoxy, the greatest value being obtained at a 10 g content of extraction diluent. On the other hand, the highest value of tensile strength was obtained at 5 g of diluent in compounds based on Epidian 53 epoxy resin. At the lowest (1 g) and at the greatest (10 g) amounts of extraction diluent, limited values of tensile strength were recorded, these values remaining above that measured on the pristine Epidian 53 resin. 

The results of the last part of the investigation can be summarized as follows: (i) the addition of the selected diluents in both epoxy resins produced general improvements in their tensile strength; (ii) the effect of the diluent content on tensile strength did not follow a precise trend; and (iii) not always did the compounds realized with the most resistant resin (i.e., Epidian 53) develop higher strength values than those produced starting from the less resistant epoxy (i.e., Epidian 5).

## 4. Discussion

From the wide and attentive investigation described so far, it was possible to conclude that the different diluents under analysis were able to affect to a certain extent the mechanical characteristics (measured in compression and tensile mode) of two commercial epoxy resins, a standard bisphenolic epoxy (Epidian 5) and a styrene-modified epoxy resin (Epidian 53), in which they were added in amounts never exceeding 10 g per 100 g of resin. The mechanical characteristics analyzed, i.e., modulus and strength, were generally enhanced upon the addition of diluents, irrespective of their chemical nature and added amounts, with the exception of compressive modulus. According to [46], if a non-reactive diluent, such as a solvent, is added to the epoxy resins, a general reduction in mechanical, chemical and adhesive properties will be observed. The observed results, therefore, suggest that some chemical interactions have occurred between the diluent and the resin or the hardener.

Compressive modulus, on the other hand, was differently influenced by the introduction of a diluent depending on the kind of resin and the type and content of diluent. A decline in compressive modulus was observed when epoxy, bituminous and nitro diluents were added to the resin initially characterized by a higher value of compressive modulus, i.e., Epidian 5 epoxy resin; only acrylic and extraction diluents, added at greater contents, led to an increase in the modulus measured in compression mode. 

The effect of the different diluents was even less clear in the case of the compounds based on the styrene-based epoxy, i.e., Epidian 53; for this resin, however, a positive effect of the different diluents on compressive modulus was generally observed at the lowest diluent content, i.e., 1 g, with the exception of epoxy diluent.

As for all the measured mechanical characteristics, i.e., compressive modulus and strength and tensile modulus and strength, no precise trends can be attributed to the amount of added diluents. Referring to their chemical nature, it was noticed that the highest value of compressive strength (i.e., 117 MPa) was obtained upon the addition of nitro diluent in the lowest amount to Epidian 5 epoxy resin (Figure 12). This diluent contains ethyl acetate, a polar aprotic solvent with a fairly high dipole moment (1.78 D), polarity probability being transferred to the diluent [47], thus improving its interactions with the resin/hardener couple. The second highest strength (i.e., 110 MPa) was measured on the compound based on the same Epidian 5 resin containing the epoxy diluent at the lowest content: the high strength value also in this case could be attributed to the high affinity of the resin and the diluent, which have the same chemical nature. 

It must be emphasized, however, that the optimal value of the mechanical characteristics that an epoxy resin/adhesive must possess depends on the specific application. Furthermore, alongside the characteristics analyzed, the effect that the presence of a diluent may have on the glass transition temperature of the epoxy resin and on its functional characteristics must be taken into account to identify the most suitable composition for the relevant application. In this regard, it is planned to investigate the influence of the same diluents on the thermal properties of both epoxy resins, i.e., their glass transition temperature and the residual heat of cross-linking reactions that the epoxy resins could exhibit, being cold-cured [17].

The obtained results are substantially in line with those reported by Ozgul and Ozkul [6] that emphasized the dependence of the mechanical characteristics on the type of diluent, and its amount, type of resin and curing agent. Most of the papers focused on the effects of diluents on the mechanical characteristics of epoxies report a gradual reduction in (compressive, flexural and storage) modulus and ultimate strength upon the addition of increased amounts of diluents (Khalina et al. [29], Kregl et al. [7] Ozgul and Ozkul [6]), the type of epoxy also being very influential. However, as Sinha et al. [33] pointed out, the addition of diluents negatively influenced the tensile strength at contents above 10 wt%. The objective of this study was, instead, to analyze the effect of limited diluent contents on the mechanical properties of epoxy resins.

From what was just outlined, it can be concluded that the research on this topic must be continuously updated because of the variety of resins, hardeners and new diluents that can be continuously proposed for specific applications.

## 5. Conclusions

In the present paper, the results of a mechanical investigation performed in compression and tensile mode on two epoxy resins, cold-cured by a polyamide curing agent, upon addition of different diluents, were analyzed. The epoxy compounds were based on Epidian 5 (a bisphenolic epoxy) and Epidian 53 (an epoxy modified with styrene) resins that were modified with epoxy, bituminous, nitro, acrylic and extraction diluents added into the epoxies in different amounts, never greater than 10 g per 100 g of each resin. The cylindrical or dog-bone specimens of the resulting epoxy compounds, cold-cured at 22 °C for two weeks, were subjected to compressive and tensile tests in accordance with ISO 604 and ISO 527-1 standards, respectively. 

On the basis of the analysis of the results of mechanical tests, the following conclusions can be drawn.

The effect of the different diluents on the compressive modulus of the epoxy resins under investigation was found to depend on the kind of resin, on the added diluent and on its content: it was not possible to identify a general trend valid for all systems. In the case of the epoxy compounds based on Epidian 5 epoxy resin, characterized by a higher value of compressive modulus in its unmodified formulation, the addition of diluents caused a decrease in this characteristic, with the exception of acrylic and extraction diluents when added in large amounts. When analyzing the epoxy compounds based on Epidian 53 resin, i.e., the styrene-based epoxy displaying a lower initial compressive modulus, increases in Ec value with respect to unmodified resin were recorded upon the addition of epoxy diluent in the largest amount (i.e., 10 g), bituminous diluent up to 5 g and nitro, acrylic and extraction at the lowest amount (i.e., 1 g).

More clear was the resulting effect of the same diluents on the compressive strength of both epoxies: their addition produced, in fact, general improvements in this characteristic, especially for the bisphenolic Epidian 5 resin at the lowest (1 g) diluent content. Epidian 53 epoxy resin, on the other hand, displayed the highest compressive strength upon addition of 5 g of nitro diluent. 

Referring to modulus and strength measured in tensile tests, both properties were generally improved with the addition of diluents. The initial characteristics of the unmodified resins mostly influenced the tensile modulus of the epoxy compounds containing diluents, i.e., greater values of tensile modulus were recorded on systems based on the stiffer (Epidian 5) resin, irrespective of the type and content of the diluent; on the other hand, the tensile strength developed by the different compounds did not always reflect the strength value offered by the resin with which they were made. Referring to the quantity of diluent added to each resin, the influence of this parameter on the tensile strength was not clear; the greatest tensile modulus values were recorded with the addition of low quantities (1 g and 5 g) of diluent, even though this was not a rule respected in all cases. As a continuation of the study, the influence of the same diluents on the thermal properties of both epoxy resins will be investigated, in particular their glass transition temperature and the residual heat of the crosslinking reactions that the epoxy resins could exhibit, being cold-cured.

The results of this study allow us to identify the type of diluent and its optimal quantity to obtain epoxy adhesives/components with mechanical properties suitable for a specific application.

## Figures and Tables

**Table 1:** Density and designations of the diluents under study.

Name of Diluent	Properties	Designation
	Density, kg/dm^3^	
Epoxy diluent	0.84	REP
Bituminous diluent	0.72	RB
Extraction diluent	0.86–0.87	REK
Nitro diluent	0.80	RN
Acrylic diluent	0.86–0.87	RA

**Table 2:** Compositions and labels of epoxy compounds based on Epidian 5/PF 100:50.

No.	Designation of Epoxy Compound	Proportions: Resin/Curing Agent/Diluent
1	EP5/PF/ERP	100:50:1
2	EP5/PF/RB	100:50:1
3	EP5/PF/RN	100:50:1
4	EP5/PF/RA	100:50:1
5	EP5/PF/REK	100:50:1
6	EP5/PF/ERP	100:50:5
7	EP5/PF/RB	100:50:5
8	EP5/PF/RN	100:50:5
9	EP5/PF/RA	100:50:5
10	EP5/PF/REK	100:50:5
11	EP5/PF/ERP	100:50:10
12	EP5/PF/RB	100:50:10
13	EP5/PF/RN	100:50:10
14	EP5/PF/RA	100:50:10
15	EP5/PF/REK	100:50:10
16	EP5/PF	100:50

**Table 3:** Compositions and labels of epoxy compounds based on Epidian 53/PF 100:50.

No.	Designation of Epoxy Compound	Proportions: Resin/Curing Agent/Diluent
1	EP53/PF/ERP	100:50:1
2	EP53/PF/RB	100:50:1
3	EP53/PF/RN	100:50:1
4	EP53/PF/RA	100:50:1
5	EP53/PF/REK	100:50:1
6	EP53/PF/ERP	100:50:5
7	EP53/PF/RB	100:50:5
8	EP53/PF/RN	100:50:5
9	EP53/PF/RA	100:50:5
10	EP53/PF/REK	100:50:5
11	EP53/PF/ERP	100:50:10
12	EP53/PF/RB	100:50:10
13	EP53/PF/RN	100:50:10
14	EP53/PF/RA	100:50:10
15	EP53/PF/REK	100:50:10
16	EP53/PF	100:50

## Data Availability

Not applicable.
